# Safety and immunogenicity of conjugate vaccine for typhoid (Vi-DT): Finding from an observer-blind, active-controlled, randomized, non-inferiority, phase III clinical trial among healthy volunteers

**DOI:** 10.1080/21645515.2023.2301631

**Published:** 2024-01-08

**Authors:** Dipesh Tamrakar, Pranodan Poudel, Pragya Thapa, Srijana Singh, Amit Khadgi, Sameera Thapa, Rajendra Tamrakar, Anmol Shrestha, Surendra Madhup, Ganesh Kumar Rai, Birendra Prasad Gupta, Tarun Saluja, Sushant Sahastrabuddhe, Rajeev Shrestha

**Affiliations:** a Dhulikhel Hospital, Kathmandu University Hospital, Dhulikhel, Nepal; b Kanti Children Hospital, Kathmandu, Nepal; c International Vaccine Institute, Seoul, Republic of Korea

**Keywords:** Typhoid, conjugate vaccine, observer-blind, randomized, phase III clinical trial

## Abstract

Typhoid fever is a significant public health concern with most of the sufferers between 15 and 25 y of age in Nepal. We undertook this study to demonstrate Vi polysaccharide conjugated with diphtheria toxoid (Vi-DT) conjugate vaccine which is non-inferior to Typbar typhoid conjugate vaccine, a Vi polysaccharide vaccine conjugated with tetanus toxoid (Vi-TT) with a focus on the adult population from Dhulikhel Hospital which was one of the total four sites in Nepal. In this study, we assigned the eligible participants in 1:1:1:1 ratio by block randomization, and stratified into three age groups (6 months to less than 2 y, 2 y to less than 18 y, and 18 y to 45 y), allotted to Group A, B, C, and D. Group A, B, and C received 25 μg (0.5 mL) of Vi-DT study vaccine and participants in Group D received 25 μg (0.5 mL) Vi-TT vaccine. We descriptively analyzed safety in all the participants receiving one dose of the investigational vaccine. The anti-Vi-IgG seroconversion rate in Vi-DT recipients was 99.71% (97.5% CI 98.04–99.96; 344 of 345 participants) and 99.13% (94.27–99.87; 114 of 115) in Vi-TT recipients which indicates that Vi-DT vaccine is non-inferior to Vi-TT vaccine. In safety aspect, 16.81% of total subject had at least one solicited adverse reaction and 22.61% of the Vi-TT participants experienced at least one solicited adverse reaction with most of them being local adverse reactions. None of the enrolled participants reported serious adverse events. Our study shows that a single dose of the Vi-DT vaccine is immunogenic, safe to administer and non-inferior to the Vi-TT vaccine four weeks after vaccination.

## Introduction

Typhoid fever is one of the major public health issues in low-resource settings like Nepal, where there is poor sanitation and hygiene practice.^1–3^ South Asia alone bears more than 70% of the global typhoid fever burden.^3^ Typhoid infection mostly affects toddlers and children aged 6 to 12 y.^4,5^ However, the recent trend shows the shift of typhoid burden toward older age group in Nepal and India with the age of most of the Nepalese typhoid sufferers ranging from 15 to 25 y.^6–8^ In the last decade, we have observed the rise in multidrug-resistant typhoid infections in Nepal along with other Asian countries as well as the emergence of extensively drug-resistant typhoid infections in Pakistan which has a direct impact on the control aspect of the disease and threatens to slow down or reverse the progress achieved in typhoid prevention and control so far.^9–14^

Typhoid fever can be prevented by providing access to safe drinking water, proper sanitation and hygiene (WASH).^15^ However, there exist significant challenges in setting up a large amount of infrastructure and maintenance of behavioral changes for easy availability of those preventive measures. The World Health Organization (WHO) recommended the systematic use of 0.5 mL single dose of typhoid conjugate vaccine (TCV) in children from 6 months and in adults up to 45 y in endemic regions in March 2018,^16^ which can also be an interim solution to limit the economic burden of the disease and combat the associated antimicrobial resistance.^17^ The 2018 WHO position paper recommends that countries should consider typhoid vaccination in high-risk groups and for outbreak control. To address the typhoid vaccine supply and demand gap, a typhoid Vi polysaccharide-diphtheria toxoid (Vi-DT) conjugate vaccine development effort was undertaken to achieve WHO prequalification and contribute to the global supply of TCV.^18^ Children below two years of age are in need of early intervention through typhoid vaccination.^19^ Controlling typhoid fever in endemic countries would necessitate immunizing young children (those under the age of 15) and including the typhoid vaccine in the schedule of the Expanded Program on Immunization (EPI).^20,21^ The introduction, wide availability and easy accessibility of suitable typhoid vaccines through mop-up campaigns is also warranted to address the burden of typhoid fever in the older population and the children missing vaccination through EPI.^22^

The WHO-prequalified Vi polysaccharide vaccine is recommended for the aged group two and above. Re-vaccination is recommended every three years owing to the limited lifespan of protective immunity.^16^ However, native polysaccharide antigens do not induce T-cells; thus, they lack the development of immunologic memory, and this limitation of Vi polysaccharide vaccines against *Salmonella typhi* is overcome by incorporation of the carrier protein to the Vi through conjugation.^23^ In children aged 9 months to 16 y, a single dosage of typhoid conjugate vaccine (TCV) is immunogenic and effective in lowering S. Typhi bacteremia which showed a reduction in the incidence of typhoid fever by 81.6% in a phase III study in Nepal using Typbar TCV, a Vi-polysaccharide tetanus-toxoid (Vi-TT) conjugate typhoid vaccine.^24,25^

The collaborative effort of the International Vaccine Institute and SK Bioscience has developed the Vi-polysaccharide diphtheria-toxoid (Vi-DT) conjugate typhoid vaccine with the aim of achieving WHO prequalification and contributing to the global vaccine supply which will address supply and demand gap created after WHO prequalification of the TCV.^16^ In phase I and II studies conducted among participants ranging from 6 months to 45 y of age in the Philippines, Vi-DT conjugate typhoid vaccine demonstrated safety and immunogenicity for both two-dose and single dose regimen.^26,27^ The phase III study was conducted in four sites: Kanti Children’s Hospital (KCH); BP Koirala Institute of Health Sciences (BPKIHS); Dhulikhel Hospital, Kathmandu University Hospital (DHKUH); and Nepalgunj Medical College (NMC) in Nepal. The study also demonstrated safety and immunological non-inferiority of Vi-DT compared to Vi-TT conjugate typhoid vaccine.^28^ As many randomized clinical trials have provided data on toddlers and children, allotting the major bulk of the study to the adult population was critical to address the scarcity of data on the safety and immunogenicity of typhoid-conjugated vaccines in Nepalese adults.^6–8,25,29^ Hence, we report age stratum specific safety and immunogenicity, mainly focusing adult age group, of a single dose of Vi-DT with a single dose of Vi-TT from subset study conducted at Dhulikhel Hospital, Kathmandu University Hospital, Nepal.

## Methods

### Study design

A randomized, observer-blind phase III study was conducted to demonstrate the non-inferiority of SK Bioscience’s Vi-DT typhoid vaccine in a single dose compared to that of Vi-TT typhoid vaccine (Typbar TCV) developed by Bharat Biotech International, India. This study was conducted at Dhulikhel Hospital, Kathmandu University Hospital along with other three different tertiary care hospitals in Nepal. The study was approved by the ethical review board of the Nepal Health Research Council (NHRC) and regulatory approval by the Department of Drug Administration (DDA). This study also received ethical approval from the Institutional Review Boards (IRBs) of the International Vaccine Institute (IVI) and the Institutional review committee of the Dhulikhel Hospital, Kathmandu University Hospital on 08 Nov 2023. Following the approval from the site ethics committee, site initiated to recruit the study participants.

### Community engagement

We achieved the optimum support and engagement of stakeholders in three phases. At first, we conducted a series of meetings with local leaders and municipal health coordinators to elaborate on the importance, rationale, and safety strategies and clear the common misconceptions prevalent among the community regarding vaccine studies. Second, we approached school headmasters and school health nurses. Study nurses and co-investigators visited the local schools and took multiple guest lectures highlighting the design and protocol of the study among school teachers, students and their guardians. Third, we invited the local media representatives to let the local press understand the nature and reality of the clinical trial and all the ethical aspects associated with it.

We regularly contacted the stakeholders throughout the study to provide updates on study progression, recruitment, and retention. At the end of the study, we once again invited all the local stakeholders to share our experience of the clinical trial as well as acknowledge and appreciate their contribution toward the successful completion of the study. These activities proved to be one of the major contributors to the smooth commencement of the first clinical trial of its nature at our site.

### Participants

The participants fulfilling the inclusion/exclusion criteria of the study were enrolled in the study. The eligibility criteria of the study were healthy volunteers aged 6 months to 45 y, participants, parents or legally authorized representatives (LARs) willing to provide consent and comply with the study procedures and not planning to leave the study area during the course of the study.

The key exclusion criteria were the presence of acute illness or any comorbid conditions, participation in any other clinical trial at the time of screening and a known history of allergy to vaccines or other medications; the factors that could impede elucidation of the study endpoints. The potential participants were pre-screened at the community and the participants and the study procedures were not started before the participants, parents or LARs provided duly signed informed written consent. A copy of the signed informed consent or assent form was provided to the participants for their records.

### Randomization and masking

The participants eligible for enrollment were assigned randomly in a 1:1:1:1 fashion into four study groups (Group A, Group B, Group C and Group D) and two study arms (experimental and comparator arm). The experimental arm of the investigational product was divided into three lots where Test Group A through Test Group C was allocated to Lot 1 through Lot 3 of Vi-DT test vaccine respectively and a single active comparator arm was apportioned to Vi-TT active control, Typbar TCV as Test Group D. The stratification was done as per the participant’s age at enrollment into one of the three age strata: six months to less than two years, two years to less than 18 y and 18 y to 45 y of age. A randomization list was generated with block sizes of four and eight by an unbiased statistician for block-randomization of the study participants aiming for a proper balance of study intervention. The masking was possible by the use of two separate randomization lists; one containing a randomization number only available to the investigator for assigning randomization number and the other containing a randomization number alongside scratch able vaccine allocation information only available to the un-blinded pharmacist and vaccinator.

### Procedures

Participants satisfying the study criteria were administered 0.5 mL (25 micrograms) of either Vi-DT vaccine or Vi-TT vaccine through intramuscular injection, preferably in the left deltoid area or the left anterolateral thigh after prior informed written consent (visit 1/visit 2). For at least 30 minutes following immunization, participants were monitored at the study site for any immediate adverse events. In a diary card, solicited adverse events were documented for 7 d after vaccination (visit 3), and unsolicited adverse events were recorded for 4 weeks following vaccination (visit 4). Serious adverse events (SAEs) were noted in a diary card and active telephonic follow-up was maintained throughout the study period of 24 weeks (visit 6). As a part of the safety evaluation, each participant’s height, weight, and vital signs were assessed at each study visit, detailed schedule of visit is described previously.^28^

Baseline blood samples from each subject were obtained prior to vaccination on the day of enrollment and two more blood samples were taken during the rest of the study period, one each on day 28 and day 168 (week four and week 24) respectively, following vaccination which was analyzed for anti-Vi IgG concentrations using in house ELISA at IVI laboratory as described previously.^27,30^ The antibody titers were measured using a reference panel from the National Institute for Biologics Standard and Control (NIBSC) which is of WHO International Standard. The value of 0.14 international units (IU) per mL served as the lower limit to detect anti-Vi IgG titers.

### Outcomes

The primary endpoint was to demonstrate the non-inferiority of the Vi-DT vaccine in terms of immunogenicity as compared to Typbar TCV® after 4 weeks (Day 28) of vaccination.

The secondary immunogenic endpoints were rate of seroconversion at 24 weeks (Day 168) of vaccination, GMT of anti-Vi IgG 24 weeks after vaccination, and secondary safety endpoints were frequency of local and systemic adverse reactions within a week of vaccination, unsolicited adverse events up to four weeks of vaccination and SAEs throughout the study duration.

### Statistical analyses

The sample size was calculated to provide 99% power for determining non-inferiority of seroconversion rate of Vi-DT vaccine (pooled groups A-C, 1350) compared to Vi-TT vaccine (group D, 450) assuming conservative 90% seroconversion and assumed non inferiority margin of −10% as per recommendation of WHO Technical Report Series 924, the details of the sample size calculation of primary study have been provided in separate publication.^28^ Non-inferiority of the Vi-DT vaccine compared to Vi-TT vaccine was considered if the lower limit of 97.5% CI for the difference between the seroconversion rates was above predefined margin. A significance level of 0.0125 was used for a one-sided test of non-inferiority. Equal number of participant were divided in four sites where DHKUH was assigned age strata 2–15 yeas (152), age strata 15–40 y (296) in numbers and additional number of 12 of age strata 6 months to 2 y were added as one of the site could recruit allocated number where BPKIHS recruited total 400, KCH recruited 480 and NMC recruited 460 of participants. The statistical analysis was performed using SAS version 9.4 and as per the protocol. The Chi-square test was used to derive *p*-values for categorical variables. Two sample t-test, the ANOVA test, and the ANCOVA test were used for the comparison of continuous variables. The safety aspect of the study was monitored by an independent data safety management board which included independent clinical experts and a biostatistician. The full analysis set (FAS), which included all the randomized participants who received one dose of the allocated vaccine, was used for demography and safety analysis. The immunogenicity analysis set, which was a subset of FAS, only included those with at least one post-baseline data on immunogenicity and was used for primary analysis of immunogenicity endpoints. The per-protocol (PP) set consisting of only those participants who did not have major protocol deviation was used to perform sensitivity analysis of primary and secondary immunogenicity endpoints. This trial is registered at ClinicalTrials.gov with the identifier (NCT number): NCT03933098.

## Results

**Figure 1.**
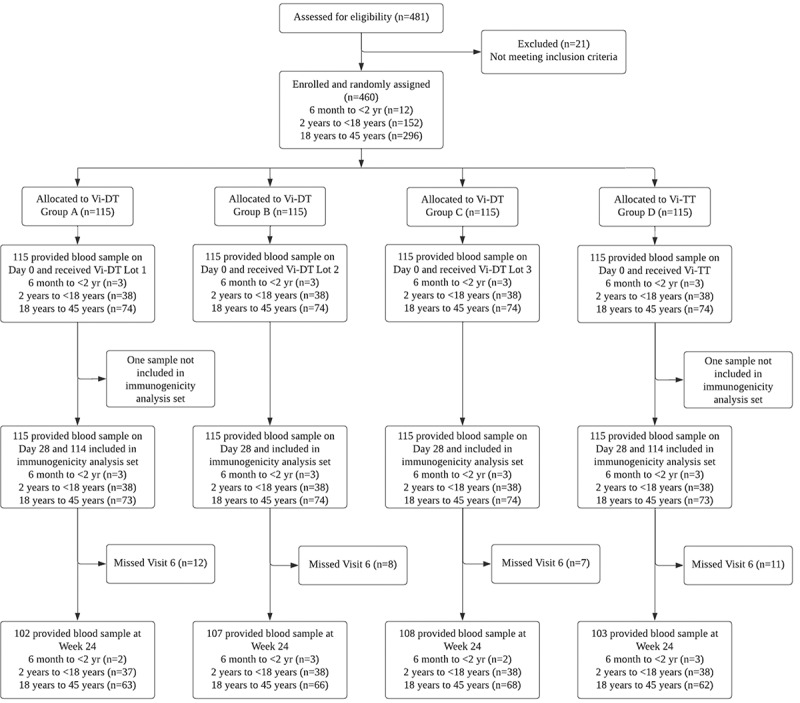
CONSORT flow diagram showing the number of patients who were screened, randomized into the treatment groups, completed the study, and included in the analysis.

A total of 481 participants were screened during the period of 29 Nov 2019 to 08 Mar 2020 of which 460 participants, 115 participants in each group; A, B, C and D, were enrolled in the study. All the enrolled participants received one dose of the allotted vaccine on Day 0 and provided one blood sample for immunogenicity on Day 28 and Day 168 for immunogenicity. We have pooled three groups (Group A + Group B + Group C) as Vi-DT groups and Group D as Vi-TT groups. The details of the participant visit, follow-up and activities performed at each visit are outlined in the flowchart as shown in Figure 1.

The mean age of the study population was 21.61 y (SD = 10.04). There were a total of 57.17% (*n* = 263) females and 42.83% (*n* = 197) males with a male: female ratio of 0.75. There were 12 participants in age strata 1 (6 months to less than 2 y), 152 participants in age strata 2 (2 y to 18 y) and 296 participants in age strata 3 (18 y to 45 y). The adult population comprised 39.53% (*n* = 117) males and 60.47% (*n* = 179) females (Table 1).

**Table 1.** Demographics information of enrolled participants.

Characteristics		Vi-DT Group	Vi-TT Group	Total	*p*-value
Overall		N = 345	N = 115	N = 460	
Gender	Male (%)	148 (42.90)	49 (42.61)	197 (42.83)	.957
	Female (%)	197 (57.10)	66 (57.39)	263 (57.17)	.831
Age (years)	Mean (SD)	21.68 (10.07)	21.39 (10.00)	21.61 (10.04)	.794
6 months to less than 2 y		N = 9	N = 3	N = 12	
Gender	Male (%)	5 (55.56)	2 (66.67)	7 (58.33)	.735
	Female (%)	4 (44.44)	1 (33.33)	5 (41.67)	.089
Age (years)	Mean (SD)	0.72 (0.09)	1.02 (0.38)	0.80 (0.23)	.044
2 to 18 y		N = 114	N = 38	N = 152	
Gender	Male (%)	59 (51.75)	14 (36.84)	73 (48.03)	.111
	Female (%)	55 (48.25)	24 (63.16)	79 (51.97)	.231
Age(years)	Mean (SD)	11.99 (3.69)	11.92 (3.79)	11.97 (3.70)	.927
18 to 45 y		N = 222	N = 74	N = 296	
Gender	Male (%)	84 (37.84)	33 (44.59)	117 (39.53)	.303
	Female (%)	138 (62.16)	41 (55.41)	179 (60.47)	.604
Age (years)	Mean (SD)	27.50 (7.10)	27.08 (7.24)	27.40 (7.13)	.663

The weight, height, heart rate, temperature, respiratory rate of all the participants and blood pressure (applicable for adults only) were similar across the study and comparator group at enrollment and during the follow-up.

The anti-Vi-IgG ELISA response was compared between the study vaccine (Vi-DT) and comparator vaccine (Vi-TT) at four weeks of vaccination. In the immunogenicity set, the seroconversion rate was 99.71% (97.5% CI 98.04–99.96; 344 of 345 participants) for the Vi-DT vaccine as compared to 99.13% (94.27–99.87; 114 of 115) for Vi-TT vaccine which demonstrated the non-inferiority of the study vaccine (*p* = .8553). The seroconversion rate for age strata 1 was 100% (70.09–100.00; 9 of 9) and 100% (43.85–100.00; 3 of 3) for the Vi-DT group and Vi-TT group, respectively. Similarly, 100% (96.74, 100.00; 114 of 114) for the Vi-DT group and 100% (90.82, 100.00; 38 of 38) for the Vi-TT group were the seroconversion rates in age strata 2. In age strata 3, 99.55% (97.49–99.92; 221 of 222) seroconverted in the Vi-DT group and 98.65% (92.73, 99.76; 73 of 74) seroconverted in the Vi-TT group (*p* = .4381). There is no significant difference in seroconversion in the two groups (Table 2). We observed similar results upon analyzing the PP set. The GMT of anti -Vi-IgG at 4 weeks for all participatns and age stratum of Vi-DT group were comparable to Vi-TT group (Table A1).

**Table 2.** Anti-vi-IgG ELISA response for seroconversion - non-inferiority data at week 4 for immunogenicity set.

	Vi-DT Group		Vi-TT Group		Difference	*p*-value
		Seroconversion rate (97.5% CI)		Seroconversion rate (97.5% CI)		
All ages	344/345	99.71 (98.04, 99.96)	114/115	99.13 (94.27, 99.87)	0.73 (−8.23, 9.69)	.8553
Age Strata 1	9/9	100.00 (70.09, 100.00)	3/3	100.00 (43.85, 100.00)	0.00 (0.00, 0.00)	NA
Age Strata 2	114/114	100.00 (96.74, 100.00)	38/38	100.00 (90.82, 100.00)	0.00 (0.00, 0.00)	NA
Age Strata 3	221/222	99.55 (97.49, 99.92)	73/74	98.65 (92.73, 99.76)	0.90 (−3.30, 5.11)	.4381

The GMT of anti-Vi-IgG at week 24 of vaccinfation for all ages was 122.06 IU/mL (95% CI 107.77, 138.25) in the Vi-DT group and 118.49 IU/mL (99.46, 141.15) in the Vi-TT group (*p*= .8723). In the participants aged 6 months to less than 2 y, the GMT of anti-Vi-IgG was 46.96 IU/mL (28.79, 76.60) in the Vi-DT group whereas 32.56 IU/mL (6.42, 165.08) in the Vi-TT group (*p* = .3732). In the age strata 2 i.e, participants aged 2 y up to 18 y, GMT was 101.84 IU/mL (86.78, 119.52) in the Vi-DT vaccine group which was significantly lower than 142.82 IU/mL (110.65, 184.35) in Vi-TT vaccine group (*p* = .0332). In the adult age group, GMT was 140.09 IU/mL (117.63, 166.85) in the Vi-DT group and 112.48 IU/mL (88.95, 142.24) in the Vi-TT group (*p* = .1379) (Table 3). The results of GMT were similar in the PP analysis set. The anti-Vi-IgG ELISA at week 24 of vaccination for all participants and in each stratum of Vi-DT group were comparable to Vi-TT group and greater than non inferiority margin of -10% (Table A2).

**Table 3.** Anti-vi-IgG ELISA GMT at week 24 for immunogenicity set.

	Vi-DT Group		Vi-TT Group		*p*-value
	N	GMT (95% CI)	N	GMT (95% CI)	
All ages	317/345	122.06 (107.77, 138.25)	103/115	118.49 (99.46, 141.15)	.8723
Age Strata 1	7/9	46.96 (28.79, 76.60)	3/3	32.56 (6.42, 165.08)	.3732
Age Strata 2	113/114	101.84 (86.78, 119.52)	38/38	142.82 (110.65, 184.35)	.0332
Age Strata 3	197/222	140.09 (117.63, 166.85)	62/74	112.48 (88.95, 142.24)	.1379

There was only one immediate reaction observed within 30 min of vaccination in the Vi-DT group. It was pain at the injection site in a participant of age group 18–45 y (*p* = 1.0000). It was moderate in nature and resolved in few hours. There were a total of 16.81% (58 of 345) participants who experienced solicited adverse events in the Vi-DT vaccine group of which 16.23% (56 of 345) were related to the vaccination while 22.61% (26 of 115) of participants experienced solicited adverse events in the Vi-TT vaccine group and only 20.87% (24 of 115) had relatedness to the vaccination. The majority of these adverse events fell into age strata 3 which accounted for 21.67% (20.72% related to vaccination) in the test group and 28.38% (25.68% related to vaccination) in the comparator group (Table 4).

**Table 4.** Solicited adverse Events.

Solicited AEs	Vi-DT Group		Vi-TT Group		*p*-value*
	Number of AEs/ Number of participants (%)	Related to vaccine	Number of AEs/ Number of participants (%)	Related to vaccine	
**Immediate reactions**					
All ages	1/345 (0.29)	1/345 (0.29)	0/115 (0.00)	0/115 (0.00)	1.0000
Age Strata 1	0/9 (0.00)	0/9 (0.00)	0/3 (0.00)	0/3 (0.00)	-
Age Strata 2	0/114 (0.00)	0/114 (0.00)	0/38 (0.00)	0/38 (0.00)	-
Age Strata 3	1/222 (0.45)	1/222 (0.45)	0/74 (0.00)	0/74 (0.00)	1.0000
**Solicited AE (Day 0 to 7)**					
All ages	58/345 (16.81)	56/345 (16.23)	26/115 (22.61)	24/115 (20.87)	.2558
Age Strata 1	2/9 (22.22)	2/9 (22.22)	0/3 (0.00)	0/3 (0.00)	1.0000
Age Strata 2	9/114 (7.89)	8/114 (7.02)	5/38 (13.16)	5/38 (13.16)	.3128
Age Strata 3	47/222 (21.17)	46/222 (20.72)	21/74 (28.38)	19/74 (25.68)	.3726

*p-value measured for AEs related to vaccination.

The solicited adverse events were reported as local and systemic adverse events (Table 5). All the local adverse events were related to the vaccination and most of the systemic adverse events were also related to the vaccination. Among the local adverse events, pain/tenderness was the most common adverse reaction 13.33% (*n* = 46) and 19.13% (*n* = 22) respectively in both the Vi-DT group and Vi-TT groups (*p* = .1293). In the systemic adverse events category in Vi-DT pooled group versus the Vi-TT group, headache 3.87% (*n* = 13) versus 4.46% (*n* = 5), respectively, was most commonly encountered (*p* = .7833) followed by lethargy, fatigue, diarrhea, nausea, loss of appetite and fever. Very few participants faced symptoms like vomiting, arthralgia, drowsiness, chills and nasopharyngitis while none experienced rash, persistent crying or irritability. Most of the solicited events were mild to moderate and resolved in few days. Two solicited event (one fever in 2 to less than 18 y group and one local tenderness in 18 to 45 y group) belonging to ViDT groups were present and resolved in few days.

**Table 5.** Distribution of solicited adverse events.

Solicited AEs	Vi-DT Group		Vi-TT Group		*p*-value^#^
	n/N (%)	m	n/N (%)	m	
**All ages**	**58 (16.81)**	**105**	**26 (22.61)**	**39**	.**1635**
**Local AE**	**47 (13.62)**	**51**	**22 (19.13)**	**22**	.**1520**
Pain/Tenderness	46 (13.33)	46	22 (19.13)	22	.1293
Erythema/Redness	1 (0.29)	1	0 (0.00)	0	1.0000
Swelling/Induration	2 (0.58)	2	0 (0.00)	0	1.0000
Pruritus	2 (0.58)	2	0 (0.00)	0	1.0000
**Systemic AE**	**22 (6.38)**	**54**	**7 (6.09)**	**17**	.**9118**
Fever	3 (0.87)	5	0 (0.00)	0	.5766
Headache^†^	13 (3.87)	14	5 (4.46)	6	.7833
Fatigue^†^	5 (1.49)	5	3 (2.68)	4	.4188
Myalgia^†^	1 (0.30)	2	1 (0.89)	1	.4379
Lethargy	7 (2.03)	7	2 (1.74)	2	1.0000
Irritability^‡^	0 (0.00)	0	0 (0.00)	0	NA
Nausea	4 (1.16)	4	2 (1.74)	2	.6427
Vomiting	2 (0.58)	2	0 (0.00)	0	1.0000
Arthralgia	2 (0.58)	2	0 (0.00)	0	1.0000
Diarrhea	4 (1.16)	4	0 (0.00)	0	.5763
Drowsiness	2 (0.58)	3	1 (0.87)	1	1.0000
Loss of appetite	4 (1.16)	4	1 (0.87)	1	1.0000
Chills	1 (0.29)	1	0 (0.00)	0	1.0000
Persistent crying^‡^	0 (0.00)	0	0 (0.00)	0	NA
Rash*	0 (0.00)	0	0 (0.00)	0	NA
Nasopharyngitis*	1 (16.67)	1	0 (0.00)	0	1.0000

N: Number of total participants; n: Number of participants who reported events; m: Number of events.

%: percentages (100*n/N).

^#^*p*-values for n/N comparisons of Vi-DT and Vi-TT groups.

*Only for MR vaccine recipients among the 9–15 months old

^†^Only for age strata 2 and 3 those who are 2 y old and above.

^‡^Only for age strata 1 those who are less than 2 y.

There were 38 instances of unsolicited adverse events reported within four weeks of vaccination in the Vi-DT pooled group from 4.64% (16 of 345) participants and for the Vi-TT group, there were 7 occurrences of such events from 2.61% (3 of 115) participants (*p* = .4280) (Table 6). All of the unsolicited adverse events were mild to moderate and resolved without sequelae. We did not observe any significant differences in the occurrence of unsolicited events in both the test and comparator vaccine groups in either age stratum. None of the unsolicited adverse events was related to the vaccination. No SAEs were reported.

**Table 6.** Unsolicited adverse events.

Unsolicited AEs	Vi-DT Group		Vi-TT Group		*p*-value*
	n/N (%)	m	n/N (%)	m	
**Unsolicited AE (within 4 weeks)**					
All ages	16/345 (4.64)	38	3/115 (2.61)	7	.4280
Age Strata 1	3/9 (33.33)	9	0/3 (0.00)	0	.5091
Age Strata 2	5/114 (4.39)	15	2/38 (5.26)	5	1.0000
Age Strata 3	8/222 (3.60)	14	1/74 (1.35)	2	.4587

N: Number of total participants; n: Number of participants who reported events; m: Number of events.

%: percentages (100*n/N).

*p-values for n/N comparisons of Vi-DT and Vi-TT groups.

## Discussion

Our study demonstrates that the Vi-DT vaccine is immunogenic and non-inferior compared to the WHO pre-qualified Vi-TT Typbar TCV vaccine which is licensed for use in Nepal, after four weeks of single intramuscular injection in all the age groups and also within each age stratum. The seroconversion rate of participants receiving Vi-DT was 99.71% which shows that the immunogenicity of the vaccine is robust.

The safety profile of the vaccine is comparable with that of Typbar TCV. Only 16.81% of the Vi-DT participants experienced at least one of the solicited adverse reactions, whereas 22.61% of the participants in the Vi-TT group faced at least a solicited adverse event and the majority of them were local adverse events, pain at the injection site is the most common one in both the pools which is also the expected outcome as the route of administration is intramuscular. There were very few cases reporting itching and swelling (0.58%) and redness over the site of injection was rare (0.29%). The occurrence of serious adverse events (SAE) was nil.

In age group 2–18 year participants, a large difference of anti-Vi IgG GMT among Vi-DT and Vi-TT groups was observed, as compared to difference observed in participants aged 6 months to younger than 2 y and those aged 18 y to 45 y. Though, the difference observed was not statistical significant, but this finding needs to be investigated further to fully understand the reasons and its clinical implications. The safety and immunogenicity data on conjugated typhoid vaccines are available from several studies which focus mainly on children and few studies recruiting the adult population had quite a small adult representation.^29,31^ A large phase III study on the efficacy of TCV was done in Nepalese children less than 16 y of age;^25^ however, the median age of the Nepalese population in a typhoid fever surveillance program was shown to be 27 y ranging mostly from 15 to 48 y of age^7,8^ which is seen as a rise in the incidence of typhoid fever in the adult population as compared to older studies.^32^ Therefore, the safety and immunogenicity data on the healthy adult participants carry a unique significance as shown by our study.

One of the limitations of the study was COVID-19-related travel restrictions, which mainly affected the follow-up visit at week 24 causing an inability to obtain blood samples from 10% of the participants from both groups; however, this had an minor impact on PP analysis. Lot to lot consistency of the different lots of Vi-DT vaccine could not be evaluated in this sub-study which has been evaluated in the main study.^28^ As our site was selected for adult and young children population, there was few number of participant in age strata 6 month to less than 2 y and also could not evaluate the immune noninterference of Vi-DT TCV with measles-rubella vaccine which has been evaluated in previously published article.^33^ Also, as Nepal is a typhoid-endemic country, there exists a possibility that few of the study participants could have a subclinical infection or they could be asymptomatic carriers. However, any such potential participants were equally distributed among four randomized groups eliminating the biased statistical findings.

In conclusion, our study demonstrates that the immunogenicity of the single dose Vi-DT TCV is similar to its WHO-prequalified counterpart employed in the study (Vi-TT TCV) and Vi-DT is also safe to administer in individuals aged six months to 45 y mainly adult population.

## Appendix

**Table A1.** GMT of anti-vi IgG ELISA response at week 4 for Dhulikhel Hospital – immunogenicity set.

Week 4 Post Vaccination	Vi-DT Group (Pooled Groups A+B+C)		Vi-TT Group		*P*-value^†^
	N	GMT* (95% CI)	N	GMT* (95% CI)	
All Ages	345	364.48 (321.84, 412.77)	115	321.30 (271.78, 379.83)	.2939
Age Strata1	9	307.35 (130.52, 723.73)	3	274.70 (94.27, 800.51)	.8071
Age Strata2	114	360.16 (302.88, 428.27)	38	475.83 (372.77, 607.39)	.0650
Age Strata3	222	369.26 (311.45, 437.80)	74	264.29 (212.74, 328.35)	.0172

*Geometric Mean Titers (unit: IU/ml).

^†^The *p*-value for all ages was derived using an analysis of covariance model with group and age strata as covariates after log transformation. The p-value for each age strata was derived from two sample t-test after log transformation.

**Table A2.** Seroconversion rate of anti-vi IgG ELISA response at week 24 for Dhulikhel Hospital – immunogenicity set.

Week 24 Post Vaccination	Vi-DT Group (Pooled Groups A+B+C)		Vi-TT Group		*P*-value^†^
	n/N	Seroconversion rate* (95% CI)	n/N	Seroconversion rate* (95% CI)	
All Ages	314/317	99.05 (97.25, 99.68)	97/103	94.17 (87.87, 97.30)	.2396
Age Strata1	7/7	100.00 (64.57, 100.00)	3/3	100.00 (43.85, 100.00)	NA
Age Strata2	113/113	100.00 (96.71, 100.00)	37/38	97.37 (86.51, 99.53)	.2517
Age Strata3	194/197	98.48 (95.62, 99.48)	57/62	91.94 (82.47, 96.51)	.0209

N: number of total participants; n: number of participants seroconverted.

*Proportion of participants who had at least 4-fold rise anti-Vi antibody titers compared to baseline.

^†^The *p*-value for all ages was derived using the generalized linear model for binomial distribution with group and strata as covariates. The *p*-value for each age strata was derived from Fisher’s exact test.
